# Development of a behavioural support intervention for e-bike use in Australia

**DOI:** 10.1186/s12889-022-14693-6

**Published:** 2022-12-21

**Authors:** Jenna McVicar, Rebecca Nourse, Michelle A. Keske, Ralph Maddison

**Affiliations:** 1 Institute for Physical Activity and Nutrition, Deakin University, Melbourne, Australia; 2 School of Exercise and Nutrition Sciences, Deakin University, Gheringhap Street, Geelong, VIC 3220 Australia

**Keywords:** Behaviour change wheel, Behavioural support, Electric bikes, Physical activity

## Abstract

**Background:**

Insufficient physical activity (PA) is a risk factor for the development of many non-communicable diseases. Electric bicycles (e-bikes) offer considerable potential to support people to be physically active, however, no previous e-bike intervention studies have supported e-bike use with behavioural support. The aim of this study was to co-develop theory-based intervention components which can be used to increase physical activity through e-cycling among people who are overweight or obese and physically inactive.

**Methods:**

We conducted a mixed-methods study using an online survey and virtual co-design workshops. We utilised the Behaviour Change Wheel (BCW) to inform the development of the behavioural support intervention to facilitate day-to-day e-cycling.

**Results:**

One hundred participants completed an online survey and seven participated in the online co-design workshops. The development of the intervention identified five intervention functions (enablement, training, environmental restructuring, education, and persuasion) and 16 behaviour change techniques (BCTs) from 11 BCT groups (goals and planning, feedback and monitoring, social support, shaping knowledge, natural consequences, comparison of behaviour, associations, repetition and substitution, comparison of outcomes, antecedents, and self-belief).

**Conclusion:**

To our knowledge, this is the first study to combine co-design and the BCW to develop a comprehensive behavioural support intervention for e-bike use. Theory based intervention options should be considered when providing e-bikes to individuals to help them increase their habitual PA levels.

**Supplementary Information:**

The online version contains supplementary material available at 10.1186/s12889-022-14693-6.

## Background

Insufficient physical activity (PA) is a risk factor for poor cardiorespiratory health, obesity, and chronic diseases such as type 2 diabetes [1–3]. The World Health Organisation’s global action plan on PA [4] specifies the importance of creating active environments to promote PA levels and the International Society for Physical Activity and Health’s eight investments for PA also focus on active travel [5]. Active travel refers to the use of walking, cycling or any other form of travel, which requires energy expenditure made by skeletal muscle. Active travel can be integrated into individuals’ day-to-day lives, offering potential to innately increase overall activity levels. People who use walking and cycling for active transport are 76% more likely to meet PA recommendations than those who use motorised transport [6]. However, only 18% of adults in Australia cycle regularly [7]. A systematic review and meta-analysis assessed the best way to promote cycling [8], researchers determined the interventions targeting cycling behaviour had a small but positive effect on cycling and noted that self-monitoring behaviour had a significant effect on cycling promotion [8]. Authors reported interventions which restructured the physical environment (e.g. built bike paths) were less effective than interventions which did not. These findings are contrary to Boufous, et al. [9] who determined improved cycling infrastructure was a facilitator to cycling promotion [9]. Furthermore, reported barriers to cycling included the travel distance being too far, challenging topography, and not being fit enough [9].

In recent years, electric bicycles (e-bikes) have emerged as a promising approach for supporting people to be physically active [10]. E-bikes are conventional bikes with battery-powered pedal assistance that supports forward motion but still require the user to pedal [11]. Compared with conventional cycling, e-bikes help individuals cycle further and for longer periods of time [12], potentially increasing the number of journeys that can be completed for recreation or for active travel. Furthermore, people have experienced increased levels of enjoyment during e-cycling compared to conventional cycling [13], demonstrating that e-bikes may be an acceptable tool for encouraging PA.

Although electrical assistance is provided, evidence suggests that e-cycling contributes to meeting PA recommendations [14, 15] and increases physical fitness among people who are inactive [16]. Our recent systematic review and meta-analysis assessed the difference in physiological responses between e-cycling, conventional cycling and walking [14]. We found a mean heart rate difference of − 11.41 beats per minute (BPM) (95% CI -17.15, − 5.68, *P* < 0.0001) between e-cycling and conventional cycling in favour of conventional cycling. Compared to walking, e-cycling with a moderate assistance level elicited an increased heart rate response (10.38 BPM, 95% CI -1.48, 22.23, *P* = 0.09). Furthermore, there was less than one metabolic equivalent (MET) difference between e-cycling with a moderate assistance level and conventional cycling, − 0.83 METS (95% CI -1.52, − 0.14), *P* = 0.02 [14].

Despite the potential benefits of e-cycling, previous interventions have provided participants with e-bikes and instruction on e-bike utilisation but did not provide any specific behaviour change support throughout the intervention period [16, 17]. Previous research has shown that theory-based interventions are more effective in promoting PA than non-theory-based interventions [18, 19]. Additionally, interventions designed with end-user input are perceived as acceptable [20]. Utilising a co-design methodology allows us to design an intervention alongside potential end users which will target specified concerns [21]. The aim of this study was to co-develop theory-based intervention components which can be used to increase PA through e-cycling among people who are overweight or obese and physically inactive.

## Methods

### Study design and context

A mixed-methods study was conducted in Australia from September 2020 to June 2021. We used an online survey and virtual co-design workshops and applied the Behaviour Change Wheel (BCW) [22] to inform the development of a behavioural support intervention for e-cycling. The BCW is a meta-framework that reflects a synthesis of 19 frameworks of behaviour change. At the centre of the BCW is a behaviour system involving three essential conditions for behaviour change: one’s capability (C) (physical and psychological), opportunity (O) (social and physical) and motivation (M) (reflective and automatic) interact to produce or change behaviour (B) (COM-B) [22]. Furthermore, the BCW includes intervention functions, policy strategies, and behaviour change techniques (BCTs) which can be applied to influence behaviour. The BCW can be used to systematically design and develop theory-based behaviour change interventions [22–25] and has been used alongside co-design methodologies [26] to further promote acceptance of the resulting intervention.

### Participants

Participants were recruited to the survey and workshops via targeted social media advertising. Our inclusion criteria for the survey and the workshops were adults (aged over 18 years), overweight (self-identified), not regularly exercising at the time of the study i.e., did not meet the physical activity guidelines and currently living in Australia. All participants provided informed consent to participate in the study. This study was approved by Deakin University Low Risk Human Ethics (reference HEAG-H 116_2020), and was conducted in accordance with the Declaration of Helsinki.

## Materials and processes

### Online survey

We conducted an online survey consisting of 25 questions (Supplementary file 1) to elicit responses on the following topics: reasons for not exercising (social and personal influences), perceptions of e-bikes, facilitators to PA and e-cycling and barriers to PA and e-cycling. The survey was hosted on Qualtrics (Qualtrics, Provo, Utah, USA) from September 2020 to November 2020. Sociodemographic information, including sex, age, self-identification of overweight or obesity, and physical inactivity status, were also collected via the survey. On completion of the survey, participants were asked to indicate their interest in participating in two virtual co-design workshops. Participants received a $10 voucher on completion of the survey.

### Virtual co-design workshops

Participants were recruited from the survey and from social media advertising. Virtual co-design workshops were hosted online (March 2021 – June 2021) via Zoom (Zoom Video Communications, Inc) by the group facilitator (JM). Participants were split into two groups, and each group attended two workshops. Participants received a $20 voucher for each workshop attended.

#### Workshop 1

In workshop 1, we discussed in-depth the barriers and facilitators to e-bike use and explored possible solutions to encourage future e-bike use. The discussion focussed on the following topics: Perceptions and understanding of PA and e-bikes, perceptions of how an e-bike might promote daily PA, and what factors and support mechanisms might facilitate e-bike use. After workshop 1, JM compounded the findings from the survey and workshop 1 and created a list of potential solutions.

#### Workshop 2

During the second workshop, the facilitator (JM) presented the potential solutions derived from the survey and workshop 1 to the participants of workshop 2. Participants were then asked to discuss the ideas and confirm that the proposed intervention components aligned with their suggestions from the first workshop.

In both workshops, an online workspace, padlet (padlet.com), was used to record ideas. As well as participating in verbal discussion, participants were asked to write their thoughts and ideas on the workspace, responses were anonymous, and participants could ‘like’ notes posted by each other. The workshop facilitator also synthesised participant discussion by adding notes to the workspace during each session. Workshops were audio recorded but not transcribed.

### Data analysis/use of the BCW by the research team

**Fig. 1:**
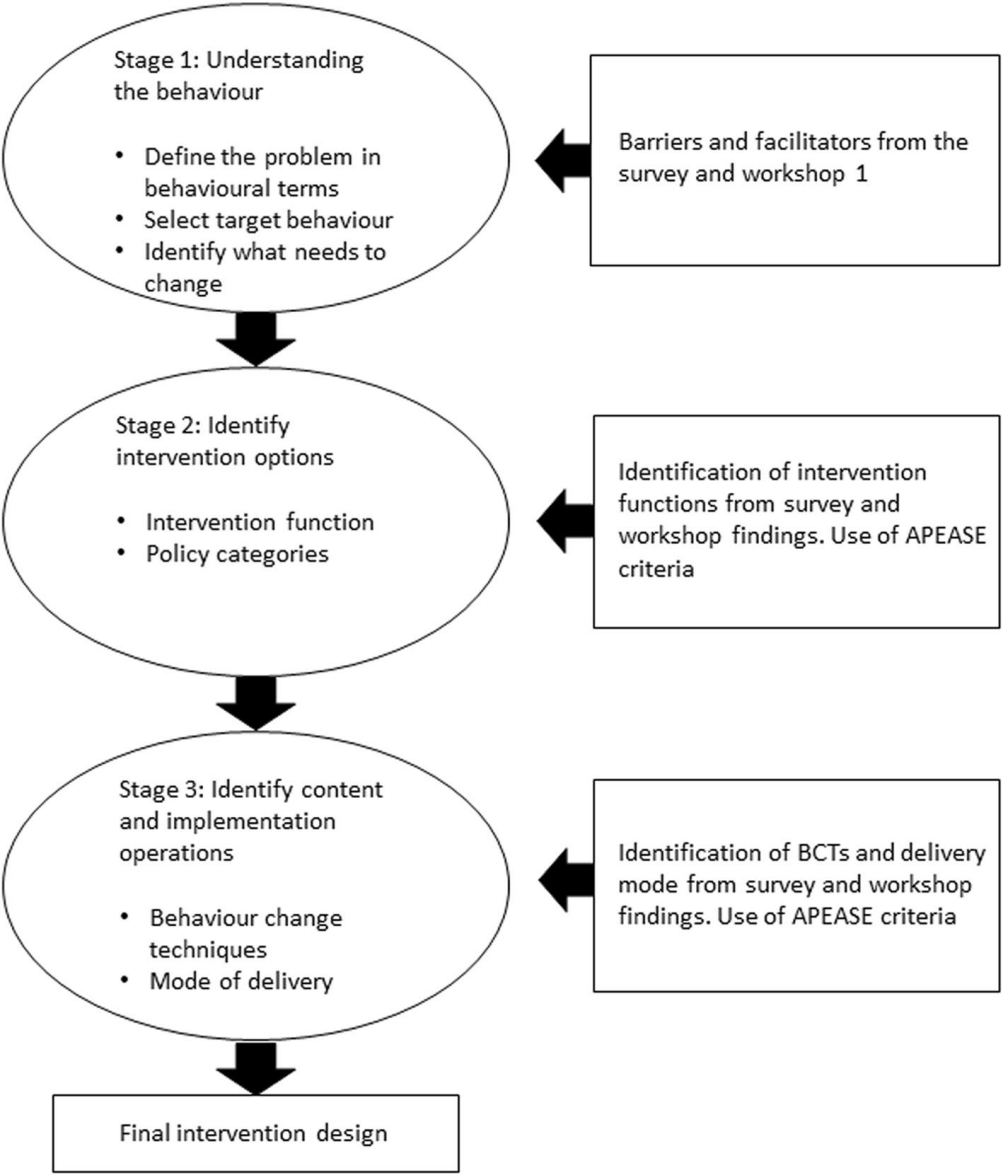
Behaviour change intervention design process [22]

The dataset analysed in this study consisted of survey results, researcher notes taken during and after the workshops, and participants’ material from the online interactive workspaces. These data were analysed according to the three stages recommended in the BCW guide [22] (see Fig. 1).

#### Stage one: understanding the behaviour

##### Steps 1–3

As outlined in the introduction, physical inactivity represents a public health concern, and e-bikes offer potential to overcome some of the barriers to participating in PA. Therefore, the target behaviour for this intervention was to increase habitual PA levels. Our goal for the remaining steps in the design process (steps 4–8) was to determine what behavioural support could be implemented alongside the provision of e-bikes. Table 1 provides an overview of the target behaviour specification.

**Table 1:** Identification of target behaviour [22]

***What target behaviour?***	Increasing PA via e-bike use
*Who needs to perform the behaviour?*	People who are overweight or obese and physically inactive
*What does the person need to do to have the preferred outcome?*	Use an e-bike to replace motorised transport for short trips or use the e-bike recreationally
*When will they perform the behaviour?*	When they can and when it is convenient for them to do so e.g., replacing short car trips
*Where will they perform the behaviour?*	Local cycle paths, parks, cycle lanes, roads

##### Step 4: identifying what needs to change

To identify what needs to change to meet the target behaviour, JM analysed the survey results and data from workshop 1 to determine which facilitators and barriers to physical activity and e-bike use were important for our target population.

#### Stage two: identifying intervention options

##### Steps 5 and 6

JM and RN identified the corresponding intervention functions utilising the APEASE criteria (affordability, practicability, effectiveness and cost effectiveness, acceptability, side-effects/safety and equity) [22] that could support behaviour change from workshop 2. The chosen functions were coded against the COM-B domains determined as relevant to target during the behavioural diagnosis. The chosen functions were selected based on evaluation against the APEASE criteria. Policy categories could be addressed in the future however were unable to be addressed within intervention components of this intervention as the research team does not have access to policy levers.

#### Stage three: identifying content and implementation options

##### Steps 7 and 8

JM and RN identified BCTs which would be most relevant for the proposed intervention. BCTs were chosen by selecting the most appropriate BCT to address what was ascertained from the survey and workshops. BCTs are the observable, replicable components of behavioural interventions and are classified as the ‘active ingredient’ of interventions [27]. The identified BCTs were discussed with the research team until a consensus was achieved. The authors discussed the most appropriate mode of delivery for each BCT, e.g., face-to-face training or an online support group. The APEASE criteria were applied when selecting the relevant BCTs and the most appropriate mode of delivery.

## Results

### Participant characteristics

In total, 302 people indicated interest to participate in the survey, 100 participants met the inclusion criteria and completed the survey. Forty-six people indicated interest to participate in the workshop, 16 met the inclusion criteria and seven contributed to the co-design workshops. Of the 100 survey participants, 60% identified as female and 39% as male, one participant did not respond. All participants included in the workshops were female.

### Intervention development

All COM-B components were considered relevant to our target behaviour.

### Intervention function

We selected the appropriate intervention functions and BCTs. Five intervention functions were identified as most likely to support the desired behaviour change: training, environmental restructuring, enablement, education, and persuasion (see Table 2). Training included upskilling on how to use the e-bike’s features e.g., an odometer to choose assistance level, charging the battery, and locking the e-bike securely. Social and physical environmental restructuring were considered important for facilitating e-bike use. Socially, participants expressed they would welcome peer support when e-cycling. Environmental restructuring included having the e-bike easily accessible, along with the required equipment e.g., helmet and charger. Enablement included providing resources to enable e-cycling such as explaining the cycle function on Google Maps (Google Inc.) to allow for access to safe cycle routes, providing cycling ponchos to protect the rider in certain weather conditions. Education included understanding the health benefits that can arise from regular e-cycling and persuasion included communicating with the participants and advising them of the previously reported positive e-bike experiences. Table 2 presents the link between the COM-B model, what needs to change, the function and evidence to support the change.

**Table 2:** Link between COM-B model, what needs to change, the BCW function and supporting evidence

COM-B	What needs to change?	BCW Function & definition	Example from data collected
Physical capability	Ensure have the physical skill to maintain e-bike Ensure have the physical skill to operate e-bike Ensure have the physical strength to maintain e-bike Have physical stamina to operate e-bike	Training: Imparting skills Enablement: Increasing means/reducing barriers to increase capability or opportunity	*“My main concern is safety in traffic. I’m very uncoordinated. I think I would be a bit dangerous- lack of spatial awareness. I’m not sure there is anything that can be done about that”*
Psychological capability	Operational maintenance of e-bike e.g., pump up tyres Understand how to operate the e-bike (e.g., assistance levels, lights) Understand how to maintain day-to-day running of e-bike (maintenance) Improve ability to remember to use e-bike Ensure have access to evidence about the benefits of using an e-bike Ensure have access to evidence about the benefits of PA Ensure have access to feedback on use of bike and health	Education: Increasing knowledge or understanding Training Enablement	*“Knowing more about how battery works, would it run out and how could I charge it etc”* *“Feedback on how much it is adding to my fitness”*
Physical opportunity	Provide e-bike to ride Provide resources to overcome weather conditions Have e-bike easily accessible to take on rides Provide resources for enablement easily accessible– pumps, helmet Ensure easy access to maintenance facilities i.e., bike shop Provide/teach access to maps to plan cycle route Need to find opportunity for PA implementation throughout the week.	Enablement Environmental restructuring: Changing the physical or social context	*“More time in the day!”* *“The cost is too high”* *“Not having many places to ride around the neighbourhood safely”*
Social opportunity	Create facilitating social environment with representation of the population group Seeing others like themselves /connecting with others e-cycling or being physically active Create supportive home environment which enables PA	Environmental restructuring Enablement	*“Support either online or from a buddy”*
Reflective motivation	Deciding to participate in PA Make plans to ride an e-bike Set PA goals Set specific e-bike use goals Move from contemplation to action of e-bike use Move from contemplation to action of PA	Education Persuasion: Using communication to induce positive or negative feelings or stimulate action	*“An app with cycling maps distances and how many kms would be good”*
Automatic motivation	Promote a sense of satisfaction after e-cycling Promote a sense of satisfaction from PA Monitor emotional reactions to PA Care more about the negative consequences of not doing it Have a strong sense that I should do it	Persuasion Enablement Environmental restructuring	*“Provide a log book to record levels of activity and experiences; and feelings”*

Incentivisation (creating an expectation of reward), coercion (creating an expectation of punishment or cost), restriction (using rules to reduce the opportunity to engage in the target behaviour, or to increase the target behaviour by reducing the opportunity to engage in competing behaviours) and modelling (providing an example for people to aspire to or imitate) were not utilised within this intervention as they were not considered suitable. Participants expressed they were not interested in a gamified incentivisation e.g., earning points or stars for PA completed, furthermore restriction was not deemed suitable as it would not be possible to restrict motorised transport use to increase e-bike use. Coercion was not deemed acceptable for this intervention as it was inappropriate to coerce people to use e-bikes and there would be no unattractive outcome if e-bike is not used. Modelling was not practical as there would be no example for people to imitate.

### Identification of BCTs

We considered 16 BCTs from the list of 93 available to be appropriate for this intervention, which fall under the categories of goals and planning, feedback and monitoring, social support, shaping knowledge, natural consequences, comparison of behaviour, associations, repetition and substitutions, comparison of outcomes, antecedents, and self-belief. No BCTs from reward and threat, regulation, identity, scheduled consequences, or covert learning categories were included. The key BCTs agreed upon were: adding objects to the environment, restructuring the social environment, demonstration of the behaviour and feedback on behaviour. The implementation of the intervention takes various forms as increasing PA levels via e-bike use has many elements involved. See Table 3 for examples of how each of the selected BCTs could be operationalised.

**Table 3:** Selected BCTs and examples of how they could be used to support behaviour change

BCT & corresponding code	BCT definition	COM-B domain	Example
Goal setting 1.1	Set or agree on a goal defined in terms of the behaviour to be achieved	Physical opportunity, social opportunity, physical capability, psychological capability, automatic motivation	Determine a SMART weekly cycling goal (e.g., distance/time/ number of rides) for the individual e-cycling
Problem solving 1.2	Analyse, or prompt the person to analyse, factors influencing the behaviour and generate or select strategies that include overcoming barriers and/or increasing facilitators	Physical opportunity, social opportunity, physical capability, psychological capability, automatic motivation	Identify specific situations where a barrier would prevent use of an e-bike (e.g., weather, time, distance) and discuss solutions to overcome these (e.g., cycling poncho, leave 5 minutes earlier, use assistance)
Action planning 1.4	Prompt detailed planning of performance of the behaviour (must include at least one of context, frequency, duration and intensity). Context may be environmental (physical or social) or internal (physical, emotional or cognitive includes ‘implementation intentions’)	Physical opportunity, social opportunity, physical capability, psychological capability, automatic motivation	Support planning of when it would be most convenient to use the e-bike
Feedback on behaviour 2.2	Monitor and provide informative or evaluative feedback on performance of the behaviour (e.g., form, frequency, duration, intensity)	Psychological capability, reflective motivation, automatic motivation,	An odometer on the e-bike to provide real-time feedback on distance cycled
Social support (unspecified) 3.1	Advise on, arrange or provide social support (e.g., from friends, relatives, colleagues, ‘buddies’ or staff) or non-contingent praise or reward for performance of the behaviour. It includes encouragement and counselling, but only when it is directed at the behaviour	Physical opportunity, social opportunity, physical capability, psychological capability, automatic motivation	Organise to cycle with a buddy or create a support group of like-minded individuals who are new to e-cycling
Instruction on how to perform the behaviour 4.1	Advise or agree on how to perform the behaviour (includes ‘skills training’)	Physical capability, psychological capability	Provide e-bike training to individuals
Information about health consequences 5.1	Provide information (e.g., written, verbal, visual) about health consequences of performing the behaviour	Psychological capability, reflective motivation, automatic motivation	Provide information on the positive health impact e-cycling has
Demonstration of the behaviour 6.1	Provide an observable sample of the performance of the behaviour, directly in person or indirectly e.g., via film, pictures, for the person to aspire to or imitate (includes ‘modelling’)	Physical capability, psychological capability	Demonstrate the features of the e-bikes and explain significance of each element
Prompts/cues 7.1	Introduce or define environmental or social stimulus with the purpose of prompting or cueing the behaviour. The prompt or cue would normally occur at the time or place of performance	Physical opportunity, social opportunity, psychological capability, reflective motivation, automatic motivation	Place the e-bike in a location that would prompt use. E-bike accessories (e.g., helmet) should equally be placed in a location which will remind people to use the e-bike.
Behavioural practice/rehearsal 8.1	Prompt practice or rehearsal of the performance of the behaviour one or more times in a context or at a time when the performance may not be necessary, in order to increase habit and skill	Physical capability, psychological capability	Prompt use of the e-bike when going to the shops instead of using motorised transport.
Habit formation 8.3	Prompt rehearsal and repetition of the behaviour in the same context repeatedly so that the context elicits the behaviour	Physical capability, psychological capability	Continue to choose e-bike over motorised transport for short trips.
Credible source 9.1	Present verbal or visual communication from a credible source in favour of or against the behaviour	Automatic and reflective motivation	Health consequences of e-cycling provided by healthcare professional.
Restructuring the physical environment 12.1	Change, or advise to change the physical environment in order to facilitate performance of the wanted behaviour or create barriers to the unwanted behaviour (other than prompts/cues, rewards and punishments)	Physical opportunity, physical capability, automatic motivation	Ensure e-bike is easily accessible and can be used as mode of transport without any inconvenience
Restructuring the social environment 12.2	Change, or advise to change the social environment in order to facilitate performance of the wanted behaviour or create barriers to the unwanted behaviour (other than prompts/cues, rewards and punishments)	Social opportunity	Advise to spend more time with friends and family who enjoy cycling for transport or recreation
Adding objects to the environment 12.5	Add objects to the environment in order to facilitate performance of the behaviour	Physical opportunity, automatic motivation	Provide e-bike to people
Verbal persuasion about capability 15.1	Tell the person that they can successfully perform the wanted behaviour, arguing against self-doubts and asserting that they can and will succeed	Automatic and reflective motivation	Advise the person they can successfully e-cycle

SMART goals, Specific Measurable Achievable Realistic Timely

## Discussion

This study aimed to develop a behavioural intervention to support overweight or obese adults who are physically inactive to increase PA levels via e-bike use. The findings from the study resulted in five intervention functions (enablement, training, environmental restructuring, education, and persuasion) and 16 BCTs from 11 BCT groups (goals and planning, feedback and monitoring, social support, shaping knowledge, natural consequences, comparison of behaviour, associations, repetition and substitution, comparison of outcomes, antecedents, and self-belief). By targeting specific facilitators and barriers to promoting e-bike use among the target population, we were able to create salient intervention features; using the BCW we were able to link barriers and facilitators to a BCT. To the best of our knowledge, no previous research studies have supported e-bike use with a behavioural intervention. All aspects of the COM-B model, physical and social opportunity, physical and psychological capability, reflective and automatic motivation, were identified as enablers to support an increase in PA levels via e-bike use. Increasing opportunity or capability can increase motivation [22]. Increased motivation can lead people to change their behaviour by increasing their capability or opportunity to do so [22].

The BCW has been used to develop interventions to promote PA and decrease sedentary time. Ojo, et al. [24] applied the BCW to develop an intervention to overcome workplace inhibitors to breaking up sitting time. The use of qualitative interviews highlighted aspects of the BCW that could be used to interrupt and reduce workplace sitting [24]. Seven intervention functions were identified; all five found in the present study plus modelling and incentivisation. Using the APEASE criteria we rejected modelling and incentivisation, however Ojo, et al. did not apply the APEASE criteria and therefore included all intervention functions. They identified 39 BCTs, they included all identified in the present study except ‘restructuring the physical environment’. This BCT is important to our intervention, the mechanism of action associated with this BCT is ‘environmental context/resources’ which links to the e-bike itself and accompanying accessories (e.g., helmet, lock, bike pump). Mechanisms of action are a range of theoretical constructs that represent how a BCT affects behaviour [28].

Facilitators to increase PA levels via e-cycling included peer support. Participants of the study advised that beginning to take part in PA and more specifically cycling, could be a daunting experience and they would welcome peer support when starting to e-cycle. By creating a sense of community for the participants, they can discuss their rides and ask questions in a non-judgemental place as all members would have the same level of cycle experience. A quantitative study exploring the role of e-bikes ability to increase women’s access to cycling and PA expressed the need for a non-judgemental, inclusive space to ask questions regarding e-cycling [29]. Creation of an online support group, such as a closed Facebook group, could encompass social exchanges and create a space for people with a common goal to meet. A pilot study within an adolescent population utilised a Facebook group as part of a mHealth intervention [30]. Authors noted that participants had a positive view of the group, a perceived sense of support and felt the group was motivating [30]. The group would not only be supportive but also be a place to generate motivation. Intrinsic motivation is key for increasing PA levels [31], therefore, implementation of a social support group could help maintain motivation and therefore sustain an increase in PA levels. Utilisation of the APEASE criteria determined an online social support group would be acceptable, practical, and affordable.

Application of the APEASE criteria ensures equity is considered. In relation to e-bikes, inequity may arise due to the higher cost associated with purchasing an e-bike compared with a conventional bike. The cost associated with e-bike purchase was raised as a concern within our findings similar to Wild, et al. [29] who also reported e-bike cost as a barrier to uptake. Reducing this disparity by providing e-bikes to individuals, providing subsidy for purchase, or increasing the opportunity to use via e-bike share schemes, could increase accessibility to e-bikes. This could theoretically remove some barriers associated with e-bike purchase such as cost, storage, and safekeeping of the e-bikes. E-bike hire schemes are being implemented across large cities [32] and the UK Government have implemented a Cycle to Work scheme where subsidy is provided for purchase of a bike [33]. Furthermore, cycle hire schemes have the potential to increase PA levels which can have an impact on health [34, 35]. Men who utilised a bike hire scheme to increase PA levels could benefit from a reduction in ischaemic heart disease and females could benefit from a reduction in depression [35].

Our findings align with the outcomes from a scoping review, which aimed to identify the intervention functions that have been used to promote cycling [36]. Environmental restructuring, including both physical and social restructuring, had the most features which could be used to inform the design of future interventions. This was followed by education, enablement, and persuasion. These findings align with our intervention functions however, training was an important function within our intervention development due to the new and unknown features associated with e-bikes. Training as an intervention function has been underutilised when developing behavioural interventions for transport behaviour change [37]. Training is used as an intervention function within many aspects of our intervention development. We established it would be required within physical capability and psychological capability (Table 2). Providing opportunity to practically apply knowledge gained was highlighted as a facilitator within the intervention development which links to BCT ‘Instruction on how to perform the behaviour. This BCT is linked with the ‘knowledge’ and ‘skills’ mechanism of action.

### Strengths and limitations

Strengths of this study include that the behavioural intervention was theory based and was created via co-design in which the needs of the participants were met. The intervention features were discussed with the participants before the final form of the invention was developed which allowed for accuracy of the intervention and its features. A limitation of this study relates to the final intervention. While we evaluated assumptions, we did not iterate the final product. Participants were provided one opportunity to discuss the developed intervention features before the intervention was finalised. The participants of the co-design workshops were a homogenous female group, which could have restricted barriers and facilitators discussed. Furthermore, participants volunteered to take part in the study and therefore may have been motivated to change their behaviour which could have impacted findings. There is a need for real world application of the intervention to determine its efficacy alongside e-bike utilisation.

### Implications

E-bikes offer utility to promote active travel and in-turn increase daily physical activity levels. They may also facilitate initiation of PA in people who perceive barriers (e.g., time, discomfort) to be greater than benefits; however, provision of behaviour support may help promote initiation and sustained use of e-bikes. Future research is needed to evaluate the effects of a behavioural support intervention on e-bike use. We are currently evaluating the behavioural component as part of 6-week, free-living e-bike intervention.

## Conclusion

To our knowledge, this is the first study to combine co-design and the BCW for development of a comprehensive behavioural support intervention for e-bike use. This intervention should be considered when providing e-bikes to individuals to help them increase their habitual PA levels.

## Supplementary Information

**Additional file 1.**

## Data Availability

The datasets used and/or analysed during the current study are available from the corresponding author on reasonable request.
