# Supplementary material for: Development of a behavioural support intervention for e-bike use in Australia
Source: BMC Public Health. 2022 Dec 21;22:2399. doi: 10.1186/s12889-022-14693-6 (PMC9768958; doi:10.1186/s12889-022-14693-6)
Supplement: Supplementary file 1 — Additional file 1. [file 12889_2022_14693_MOESM1_ESM.docx]

Q1. I have read and understand the Plain Language Statement and agree to freely participate in this study.

- I indicate that I have read the Plain Language Statement and consent to participate (1)
- I do not want to participate (2)

Skip To: End of Survey If I have read and understand the Plain Language Statement and agree to freely participate in this s... = I do not want to participate

Display This Question:

If I have read and understand the Plain Language Statement and agree to freely participate in this s... = I indicate that I have read the Plain Language Statement and consent to participate

Q2. I am 18 years old or older.

- Yes (3)
- No (4)

Skip To: End of Survey If I am 18 years old or older. = No

Display This Question:

If I am 18 years old or older. = Yes

Q3. Do you self-identify as being overweight or obese?

- Yes (1)
- No (2)

Skip To: End of Survey If Do you self-identify as being overweight or obese? = No

Display This Question:

If Do you self-identify as being overweight or obese? = Yes

Q4. Do you currently complete 150 minutes of moderate to vigorous physical activity per week, e.g., brisk walking, running, dancing, fast cycling...?

- Yes (1)
- No (2)

Skip To: End of Survey If Do you currently complete 150 minutes of moderate to vigorous physical activity per week, e.g., br... = Yes

Display This Question:

If do you currently complete 150 minutes of moderate to vigorous physical activity per week, e.g. br... = No

Q5. Do you live in Australia?

- Yes (1)
- No (2)

Skip To: End of Survey If Do you live in Australia? = No

Q6. What is your gender?

- Male (1)
- Female (2)
- Other (Please specify) (3) ________________________________________________
- Prefer not to say (4)

|  |
| --- |

Q7. What age are you?

________________________________________________________________

Q8.a Do you currently ride a bike? (Any type)

- Yes (1)
- No (2)

Display This Question:

If do you currently ride a bike? (Any type) = Yes

Q8.b If yes, how often?

________________________________________________________________

Q8.c Have you cycled an e-bike before?

- Yes (1)
- No (2)

Display This Question:

If have you cycled an e-bike before? = Yes

Q8.d Do you regularly cycle an e-bike?

- Yes (1)
- No (2)

Display This Question:

If do you regularly cycle an e-bike? = Yes

Q8.e How often do you e-cycle?

Q9. Why are you looking to be more physically active?

Q10. What would help you to be more physically active?

Q11. What stops you from being active?

Q12. Listed are some causes that may be a reason for being active, do you agree with these statements?

|  | Strongly disagree (1) | Somewhat disagree (2) | Neither agree nor disagree (3) | Somewhat agree (4) | Strongly agree (5) |
| --- | --- | --- | --- | --- | --- |
| Being more active would help me relax (1) |  |  |  |  |  |
| Walking or cycling to work or the shops would save me time (2) |  |  |  |  |  |
| Walking or cycling to work or the shops would save me money (3) |  |  |  |  |  |
| My family influence my activity levels (4) |  |  |  |  |  |
| My friends influence my activity levels (5) |  |  |  |  |  |

Q13. Please provide a short description of what you think an e-bike is.

Q14. Do you think an e-bike would be easy to use?

- Yes (1)
- No (2)

Q15. What difficulties do you see from using an e-bike? You can choose more than one and can add below any other difficulties you think you may face.

- It would be difficult to charge. (1)
- I am worried it would run out of charge while riding. (2)
- It will be heavier than a normal bike. (3)
- It will be too fast for me. (4)
- I wouldn't feel safe while cycling an e-bike. (5)
- Other - please add below. (6)

Q16. Please add any other difficulties you imagine may occur when using an e-bike.

Q17. Do you think it would be expensive to purchase an e-bike?

- Yes (1)
- Maybe (2)
- No (3)

Q18.Would this initial cost be a reason to not buy one?

- Yes (1)
- Maybe (2)
- No (3)

Q19.
Listed are some statements associated with cycling and e-cycling, do you agree with these?

|  | Strongly disagree (1) | Disagree (2) | Somewhat disagree (3) | Neither agree nor disagree (4) | Somewhat agree (5) | Agree (6) | Strongly agree (7) |
| --- | --- | --- | --- | --- | --- | --- | --- |
| I am confident in my cycling ability (1) |  |  |  |  |  |  |  |
| I would like to purchase an e-bike. (2) |  |  |  |  |  |  |  |
| I would get healthier cycling an e-bike compared with a normal bike (3) |  |  |  |  |  |  |  |
| I would get healthier using an e-bike compared to using the car or taking the bus (4) |  |  |  |  |  |  |  |
| It would be easier to cycle an e-bike compared to a normal bike or walking (5) |  |  |  |  |  |  |  |
| I would like to use an e-bike in my daily routine (6) |  |  |  |  |  |  |  |
| I would use an e-bike to replace car trips (7) |  |  |  |  |  |  |  |
| I would use an e-bike to increase my activity levels (8) |  |  |  |  |  |  |  |

Q20. Listed are some things that may stop you from e-cycling, do you agree or disagree with these statements?

|  | Strongly disagree (1) | Disagree (2) | Somewhat disagree (3) | Neither agree nor disagree (4) | Somewhat agree (5) | Agree (6) | Strongly agree (7) |
| --- | --- | --- | --- | --- | --- | --- | --- |
| Lack of cycle lanes (1) |  |  |  |  |  |  |  |
| Feeling unsafe cycling on roads (2) |  |  |  |  |  |  |  |
| Weather (3) |  |  |  |  |  |  |  |
| Too physically demanding (4) |  |  |  |  |  |  |  |
| I am too unfit (5) |  |  |  |  |  |  |  |
| Cycle distance too far (6) |  |  |  |  |  |  |  |
| I don't have enough time (7) |  |  |  |  |  |  |  |
| I will get sweaty riding (8) |  |  |  |  |  |  |  |
| I am concerned the bike would get stolen (9) |  |  |  |  |  |  |  |
| I would have to wear special clothing to ride (10) |  |  |  |  |  |  |  |

Q21. Please state any other barriers you may have associated with e-cycling.

Q22. Listed are some things that could help encourage you to ride an e-bike. 


To what extent do you agree with the following?

|  | Strongly disagree (1) | Disagree (2) | Somewhat disagree (3) | Neither agree nor disagree (4) | Somewhat agree (5) | Agree (6) | Strongly agree (7) |
| --- | --- | --- | --- | --- | --- | --- | --- |
| Having maps of local trails and cycle routes easily available (1) |  |  |  |  |  |  |  |
| A buddy/friend to cycle with (2) |  |  |  |  |  |  |  |
| Goal setting - e.g., "I want to cycle 10km/2 hours total this week" (3) |  |  |  |  |  |  |  |
| Understanding the benefits of cycling (4) |  |  |  |  |  |  |  |
| Tracking cycling progress - e.g., time/distance cycled weekly (5) |  |  |  |  |  |  |  |
| Feedback on my fitness and health status (6) |  |  |  |  |  |  |  |
| Mobile app to provide support and encouragement to ride (7) |  |  |  |  |  |  |  |

Q23. Please state any other ideas you think would encourage you to e-cycle.

Thank you Thank you for taking part in our survey, if you are interested in taking part in the development of a behavioral intervention please advise below and state the email address you wish to be contacted on. We also require your e-mail address to send you a gift card as a thank you for taking part in the survey.

Q24. I would like to take part in the development of the behavioral intervention.

- Yes (1)
- No (2)

Q25. Please provide your details so that we can contact you for phases 2 and 3. If you have chosen not to be contacted, please provide these details to allow us to send you the gift voucher.

- Name (1) ________________________________________________
- Email (2) ________________________________________________
